# Professional Esports Players: Motivation and Physical Activity Levels

**DOI:** 10.3390/ijerph19042256

**Published:** 2022-02-16

**Authors:** Frano Giakoni-Ramírez, Eugenio Merellano-Navarro, Daniel Duclos-Bastías

**Affiliations:** 1 Grupo de Investigación EFISAL, Universidad Autónoma de Chile, Temuco 4810101, Chile; emerellano@gmail.com; 2 Escuela de Educación Física, Pontificia Universidad Católica de Valparaíso, Valparaíso 2374631, Chile; daniel.duclos@pucv.cl

**Keywords:** esports, physical activity, videogame, motivation

## Abstract

The professionalisation of esports has increased in recent years, generating the need for further study. Its evolution and continuous development have led the consideration of esports as a profession, increasing the number of players, practice modalities, and hours of play dedicated to this field. The aim of this study was to analyse the relationship between physical activity levels and motivational orientations in an international sample of professional esports players. A cross-sectional and observational study was conducted in European and Latin American countries. The sample was non-probabilistic by convenience, and 260 male professional esports players were recruited. A survey was used that included demographic data, body composition, physical activity (International Physical Activity Questionnaire), and motivation (Sport Motivation Scale). The results show that 92.7% of professional esports players have moderate and high levels of physical activity and that players with low levels of physical activity have positive values in all dimensions of motivation. It is concluded that extrinsic and intrinsic motivation correlates inversely with energy expenditure.

## 1. Introduction

Electronic sports (esports) began to gain popularity in the gaming community in the early 2000s [1], reaching an estimated mass of 395 million people around the world in 2018 [2]. The exponential growth of esports in the world [3] has led to this recreational activity being recognised as a sport [4], generating a source of employment for players who master and demonstrate skills related to the game, becoming professional players sponsored by recognised companies and with the possibility of winning large cash prizes. Progress in this area has resulted in 201.2 million consumers of esports [5], i.e., actively participating in or consuming esports events, while 1757.5 million people have heard of esports without participating in or watching it [5].

Esports refers to competitive video games in which teams or individuals compete against each other. It is considered a sporting activity in which players can develop and train their mental skills and hand-eye coordination while playing [6,7,8,9]. In recent years, esports revenues (e.g., merchandising, tournament tickets, commercial contracts with brands, media rights and sponsorships) also grew notably, reaching in total revenues in 2019 an increase of 26.7% from 2018, which is equivalent to USD 1.096 million [5].

Competitive video games comprise esports tournaments organised with rules, systems, gameplay, evaluation, and broadcasting similar to more traditional sporting events. In addition, professional esports players face similar training requirements to other sports athletes [10], so their form of organisation is similar to traditional sports, as it identifies the presence of rules, competitions, and training, among other aspects; even authors such as [11] consider esports as a new sport although participants have reduced physical activity involvement.

Moreover, it is worth noting that, in recent years, interest in esports has increased not only for gamers but also because of the enthusiasm it generates among spectators and investors [12]. Although research on video games has predominantly focused on problematic use or addiction [13,14,15], it has become a recreational activity for most people. This has led different scientific fields (e.g., marketing, law, exercise, and health) to start studying esports in more depth, making esports a fruitful topic for scientific research.

### 1.1. Physical Activity and Esports

Esports comprise a group of different classifications of video games (e.g., multiplayer arena, first-person shooter, collectible card, real-time strategy, last survivor, fighting, sports simulators, and driving games) that are played in a specific environment (i.e., online), becoming internationally and globally executed [16,17,18,19]. Esports can be run under organised, rule-governed competitions that require skill and have a wide following; however, esports currently lack regulation and institutionalisation [20].

On the other hand, and in relation to their professionalisation, only a few esports players can reach the professional level [21]. The development of this type of sport contributes to athletes reaching a high level of performance, so it is important to delve into how screen time, physical inactivity, and sedentary behaviour in these athletes could affect their health and sporting performance.

Several studies point out that physical inactivity and sedentary lifestyles are a global public health problem and have been associated with short- and long-term health risks, such as cardiovascular disease, psychological problems, and cancer [22,23]. Conversely, high levels of physical activity have been associated with reduced mortality and major comorbidities [24,25,26,27].

Some studies suggest that esports players engage in sedentary behaviours for 4.2 h per day while training [28]. The amount of time esports players spend sitting has potential negative consequences, including increased risk of injury and chronic diseases, such as upper limb dysfunction, metabolic dysregulation, circadian rhythm problems, and neck and back problems [29]. To date, there is no empirical evidence on whether professional esports players are more or less physically active than the general population and whether this relationship is associated with other negative health behaviours and obesity levels. However, on the contrary, there are studies that have reported that, as part of their training regimen, elite gamers spend 1.08 h per day exercising [28]. Other studies also claim that esports players include physical training as a strategy to improve gameplay and manage stress; however, only 6–9% of esports players report exercising for perceived performance benefits, while 32–47% engage in physical activity primarily for general health benefits [19,28]. Although both individual and team esports players report that physical activity is a component of their overall training, there is currently no evidence to indicate the effect of physical activity on players’ overall performance or general health. However, recent studies on the physical activity levels of sports simulator esports players reported regular and vigorous physical activity [19] even above international recommendations [28], which could be a driving force for improving physical activity for both esports players and spectators who follow esports [19,30]. In fact, esports players at elite levels of different videogame genres have been reported to have a BMI status of normweight, which goes against the empirical notion that esports players are obese because they are physically inactive [28,31,32]. In addition, exercise and physical activity may be important to improve esports performance [30,33].

### 1.2. Motivation

Motivation can be initially defined as the tendency of people to do something for some purpose. It corresponds to a state of energization and activation of the organism in a social context [34]. One of the psychological theories that has had an important development in the field of motivation is the Self-Determination Theory (SDT) [35]. This theory is based on the assumption that human beings tend toward self-regulation, competence, and integrated action. Motivation is seen as a multidimensional construct, which can vary not only in intensity but also in the type of motive. SDT considers that human beings are not merely conditioned by external reasons but are assumed to have volition, freedom, and autonomy [36].

Intrinsic motivation (IM) is defined as the practice of the activity in the absence of external contingencies [37]. It has been postulated that intrinsic motivation is a global construct composed of sub-types of regulation [35].

Extrinsic motivation is related to a variety of behaviours that do not have a purpose in themselves. The sport action is, in this case, a means to other ends. The reasons for behaviour in EM move on four levels, ranging from more to less self-determination. These adjacent levels of a continuum correspond to various classes of regulation: (a) Integrated regulation (INTEG) represents the most self-determined form of the internalization process and occurs when the motivational rationale is consistent and harmonious with the individual’s self-concept, other schemas, and values [38]. (b) Identified regulation (IDR) occurs when people judge the reason for a behaviour as important, and although the activity is still performed for extrinsic reasons (e.g., the achievement of personal goals), the behaviour is internally regulated [38]. (c) the Introjected regulation (INTY) corresponds to internal representations of external contingencies. (d) Finally, external regulation (EXT) corresponds to those behaviours that are controlled by external sources, such as material reinforcement or obligations imposed by others.

Amotivation (AMOT): In AMOT, feelings of self-determination are absent, and there are no extrinsic or intrinsic reasons that support its relationship with behaviour. Amotivation is similar to what is understood as hopelessness [39]. This regulation is associated with feelings of incompetence and lack of control. Non-participation or abandonment is possible [35].

### 1.3. Motivation in Esports

There are several studies that analyse the motivations for playing sports [40,41,42,43,44,45]; however, there are still few studies that analyse the motivations that lead players to play esports competitively. In this way, Seo [3] and Kim and Thomas [46] highlighted that intrinsic motivations and the acquisition of an esports player’s identity can be decisive in becoming a professional. In relation to acquiring an identity as an esports player, Seo [3] found in field observation sessions and interviews with 10 professional esports players that players, when they were from an amateur category, observe playing video games as a casual leisure activity (i.e., playing for fun) and gain interpersonal relationships within the esports community, and as they improve their skills and knowledge, esports gradually become an important aspect of their lives and identity. Furthermore, it was concluded that the main characteristics of esports players who choose competitive gaming as a career path were mastering skills; the pursuit of self-improvement; the importance of fairness, equality, and respect in the community; and experiencing high self-esteem, achievement, and recognition [3].

Kim and Thomas [46] examined how the motivations (intrinsic and extrinsic), goals, and learning style of professional esports players change during the process of becoming a professional. Following their interviews with professional esports players, their team coaches, team manager, and psychological counsellor, five different stages in the process of becoming a professional esports player were identified. The motivation pattern of the players changed during each stage. For a beginner in the esports scene, the gaming activity itself is motivating enough. By gaining more experience and struggling to win and lose, meeting more experienced opponents, and competing in video games, the games lose their fun factor. However, by developing greater competition, the enjoyment of gaming intrinsically motivates experienced esports players. Despite this, Kim and Thomas [46] drew attention to the need to distinguish casual esports players based on the change in their motivational patterns. More specifically, competing at higher levels should be considered a job and is generally driven by extrinsic motivation (e.g., tournament prizes, rewards, and fame) rather than intrinsic motivation.

On the other hand, other works have investigated different aspects of gaming motivations by comparing esports players with casual gamers. Martončik [47] highlighted that professional gamers compete in video games to satisfy their life goals (i.e., intimacy, affiliation, altruism, power, achievement, and fun). Affiliation (i.e., the need to help others, active interaction, and relating to others) differentiated professional esports players from casual players, probably because professionals tend to develop meaningful relationships with team members and other members of the esports scene. In addition, distraction motivation (i.e., the need for excitement, tension, and new experiences) also drives professional players more than casual players to compete in video games. Moreover, those professionals who were leaders of esports teams also satisfied their need for power by occupying the leadership position [47]. In a more recent study, Bányai et al. [1] concluded that professional esports players played more (more game time on weekdays and weekends) and scored higher on social motives (developing and maintaining relationships with other players), competition, and skill development than casual players. These results are consistent with research among athletes in conventional sports that has shown similar results. More specifically, the motivational pattern of sports has both intrinsic and extrinsic aspects. Traditional athletes enjoy the competition itself, internalise the identity of the professional athlete, and constantly strive for self-improvement, but they may also be motivated by extrinsic motivations, such as stimuli, rewards, or fame [40,41,42,43,44,45].

In summary, previous studies have reported that intrinsic and extrinsic motivations, such as competition, challenge-seeking, social factors, and the drive for self-development, are key motivations among professional esports players. In addition, professional gamers have different motivations compared to casual gamers, and these motivations change during their career. However, the findings of previous studies still raise the question of which motivations (intrinsic or extrinsic) are more important in the career of a professional esports player. In order to answer this question, the main purpose of this paper is to establish the relationship between physical activity levels and motivational orientations in an international sample of professional esports players.

## 2. Materials and Methods

### 2.1. Sample

A total of 260 male professional esports players [48] from 7 countries, namely Chile, Argentina, Brazil, Mexico, Spain, Germany, and Sweden, were considered for the sample of the present study, exceeding in number several researches [3,46,49]. The age range of the participants was 18–30 years (M = 21.30; SD = 2.26). Table 1 shows the sociodemographic characteristics of the participants. Inclusion criteria were as follows: (a) participants had to be members of a professional esports team; (b) agree to participate in the study by voluntarily submitting the questionnaire, which they had to complete online; and (c) sign the informed consent form authorising the use of the information for scientific purposes in accordance with the Declaration of Helsinki [50].

The information was collected between March and April 2021 using non-probabilistic convenience sampling. This sampling method was chosen because of the difficulty of carrying out a probability sample due to the lack of an updated register. In addition, the research objectives did not aim for representativeness or generalisability of the results. Finally, we had access to a database of potential subjects who met the inclusion criteria, which also justified the use of this type of sampling.

### 2.2. Instruments

Two instruments were used to obtain the information. Firstly, the International Physical Activity Questionnaire (IPAQ), a questionnaire that assesses self-reported physical activity levels, was administered. It has been validated and translated into different languages [51] and consists of 7 items that consider the time and energy expended (metabolic equivalent of the task, MET-min week) in vigorous intensity activities, moderate intensity activities, and walking, which were performed for more than 10 min in the previous 7 days.

MET levels of 3.3, 4, and 8 were assigned for walking and moderate-, and vigorous-intensity activities, respectively. Each participant was assigned one of the following three levels:

1. very active: if you engaged in vigorous physical activity on 3 or more days per week, accumulating at least 1500 MET-min per week, or 7 or more days where the combination of moderate/vigorous activity or walking accumulated at least 3000 MET-min per week);

2. moderately active: if you performed 3 or more days of vigorous physical activity per week (more than 20 min/week) or more than 5 days with moderate physical activity and/or walking (at least 30 min/day) or 5 or more days with a combination of vigorous/moderate physical activity or walking accumulating 600 MET-min week);

3. not very active: no physical activity or insufficient to meet the criteria of the very or moderately active categories [51].

The total MET-min-week variable was calculated by summing the individual MET-min-weeks for each activity intensity: vigorous, moderate, and walking. As proposed by the IPAQ scoring protocol guidelines [51], values below 10 min were recoded as “0”, and values above 960 min of physical activity were truncated to 960 min. Thus, when physical activity variables (i.e., vigorous and moderate physical activity and walking) exceeded 180 min/day, they were truncated to 180 min only. The IPAQ also includes a question on time spent sitting. One question is: “During the last 7 days, how much time (hours per day and minutes per day) did you spend sitting on a weekday?”, which was analysed separately from the questions included in the physical activity score. Finally, physical activity levels were analysed according to the recommendations on physical activity and sedentary behaviour proposed by the World Health Organization (2020). Sessions of less than 10 min of physical activity were recoded following the IPAQ scoring protocol.

Then, the Sport Motivation Scale was applied. The original scale was called Échelle de Motivation dans les Sports [37] and was translated and validated into English by Pelletier et al. [39] and renamed Sport Motivation Scale (SMS) and subsequently translated and validated into Spanish by Balaguer, Castillo, and Duda [52]. Both versions consist of 28 items that measure the different types of motivation established by the self-determination theory [35]. The instrument considers seven subscales: (1) no motivation; (2) external regulation; (3) introjected regulation; (4) identified regulation; (5) intrinsic motivation to learn; (6) intrinsic motivation as personal satisfaction; and (7) intrinsic motivation by sensory stimulation. Scales 2 to 4 correspond to extrinsic motivation and 5 to 7 to intrinsic motivation. All subscales were evaluated with a weight of 7 points, where 1 means “never”, 2 “seldom”, 3 “rarely”, 5 “sometimes”, 6 “many times”, 7 means “always”, and 4 as a midpoint means “undecided”.

Finally, the participants in the sample had to indicate their body weight (kg) and height (cm) on the form. With these data, the body mass index (BMI = kg/m^2^) was obtained, which made it possible to determine the nutritional status according to the categories, including undernutrition, normal weight, overweight, and obesity, based on the guidelines of the WHO [27] according to gender and average age and used in a previous study on body composition of professional esports players by Giakoni-Ramírez, Duclos-Bastías, and Yañez-Sepúlveda [31].

### 2.3. Procedure

First, managers and coaches of professional esports teams from different countries around the world were contacted via email to invite them to participate in the research. Those who responded favourably were asked for the e-mail addresses of the players in their respective teams.

Subsequently, a voluntary participation invitation email was sent to each player, which contained the link to the informed consent and the instrument (IPAQ and SMS-28). A total of 260 responses were received with a signed consent form and instrument via the online platform Google Forms© adhering to the Singapore Declaration [53].

### 2.4. Statistical Analysis

The statistical analysis was carried out using IBM SPSS^®^ 27 software. The characteristics of the sample (Table 1) were obtained through a descriptive analysis, obtaining central tendency statistics, such as mean and standard deviation for continuous variables and frequency distribution for categorical variables. To determine the normality of the sample, the Kolmogorov–Smirnov test was applied. Additionally, differences in motivation and levels of physical activity among professional players were analysed using ANOVA test. To determine statistical differences between the region of origin of the subjects for the dimensions of motivation, we used the Student’s *t*-test. Finally, to analyse correlations between numerical variables, Pearson’s correlation coefficient was used without adjustment for a covariate.

## 3. Results

The characteristics of the sample are presented in Table 1. A total of 63.5% of the players reported professional experience between 12 and 24 months, and only 4.6% reported more than 48 months. The specialities in which they compete is diverse, with the highest being 44.6% of participants playing League of Legends, 33.1% Counter Strike, 6.9% Call of Duty, and 8.5% Clash Royale. In terms of BMI, 49.6% of the sample was of normal weight.

Table 2 shows the results obtained on the levels of physical activity and motivation. It should be noted that 48.5% of the subjects presented high levels of physical activity, followed by 44.2% who performed moderate activity, while only 7.3% reported low levels of physical activity. As for the SMS scale scores, the highest scores were found for the IM—stimulation (M = 5.64; SD = 0.57) and identified regulation (M = 5.53; SD = 0.75) dimensions. In contrast, amotivation is the dimension with the lowest score (M = 2.21; SD = 0.48).

Table 3 presents the dimensions of motivation as a function of physical activity levels. Professional esports players with low levels of physical activity have the highest values in all SMS dimensions, with the exception of amotivation, where the distribution is inverse. The dimensions IM—stimulation, identified regulation and introjected regulation, and external regulation show statistically significant differences between physical activity levels (*p* < 0.05).

On the other hand, Table 4 presents the dimensions of motivation, BMI, METs, and sedentary time as a function of region (Europe and Latin America). Statistically significant differences can be seen in motivation (*p* = 0.02), total BMI (*p* = 0.04), and sedentary time (*p* = 0.01).

Finally, Table 5 presents the correlation coefficients between the dimensions of motivation (intrinsic, extrinsic, demotivation, and total motivation), physical activity (total METs), sedentary time, weight, height, age, and months as a professional. The results show low positive correlations between intrinsic motivation and demotivation (*r* = 0.257), low negative correlation with total METs (*r* = −0.158); low correlation between extrinsic motivation and demotivation (*r* = 0.269); and low negative correlation with total METs (*r* = −0.174). Age has a high correlation with professional months (*r* = 0.685), and finally, the variable weight correlates positively with height (*r* = 0.383).

## 4. Discussion

The purpose of this study was to find out and establish the relationship between physical activity levels and motivational orientations in an international sample of professional esports players. The main results highlight that more than 90% of esports athletes have high and/or moderate levels of physical activity; in addition, professional esports players with low levels of physical activity have positive values in the dimensions of motivation, and finally, extrinsic and intrinsic motivation of athletes correlates inversely with energy expenditure.

Esports, like traditional sports, have a physical implication [54]; in relation to this, the present study aims to be a contribution to the development of esports. The development of research in this field indicates that the preparation of esports athletes not only requires tactical analysis or the ability to respond to something that appears on the screen but also the endurance of the player to perform these activities over a long period of time.

Esports players are often considered sedentary, as the game requires prolonged physical inactivity. However, the results obtained in the present study indicate that 92.7% of the sample of professional esports players presented high and moderate levels of physical activity, registering a range of METs between 2428.55 and 2533.23. Studies that have related physical activity to other health behaviours, both with American and European samples, indicate that people who engage in some type of physical activity or sports practice, in addition to obtaining physical and psychological benefits, have healthier behaviours than physically inactive people [55,56]. These results are similar to those obtained by Paramitha et al. [49], who conducted a study with a sample of 50 professional gamers, reporting a mean of 3120.2 METs. Similarly, the findings of the present study are supported by Rudolf et al. [57], who determined that two-thirds of esports players in Germany reach the WHO physical activity recommendations. This is also in agreement with DiFrancisco-Donoghue et al. [58], who reported that around 60% of U.S. and Canadian college esports players participated in regular and pre-competition physical activity, and players reported practising between 5.5 and 10 h per day. On the other hand, Pereira et al. [59] showed that the majority of players participating in esports competitions meet physical activity recommendations. The majority of players (73%) reported high levels of physical activity, encompassing regular physical training, which was mainly focused on promoting health-enhancing physical capacity rather than improving esports performance.

In contrast, some current research on physical activity in esports players shows inconsistent results; an international online survey conducted by Trotter et al. [32] showed that 80.3% of esports players do not meet the WHO physical activity recommendations, which is not decisive for establishing a total lack of physical activity in this type of population.

In relation to BMI, 49.6% of the sample that participated in this research is normal weight, which is in line with the results recorded by Giakoni et al. [31], who conducted a study with a sample of Spanish professional esports players, allowing to determine a clear trend regarding the body composition of the subjects, stating that players have mostly a normal weight, ruling out the prevalence of overweight and/or obesity. On the other hand, the results found by Trotter et al. [32] indicated that the BMI of esports players from Germany, Canada, and the United Kingdom differed significantly from the global BMI reference data of the general population, with esports players being more likely to have their BMI classified as normal weight and pre-obese; in addition, a healthier BMI was associated with higher physical activity level and higher levels of perceived general health [32]; these results coincide with the results of the study by Trotter et al. [32] and Paramitha et al. [49], who in the analysis of a sample of 50 professional gamers, found a BMI = 22.4, which is considered normal weight. However, a study in university-level gamers [58] suggested that esports could be considered a sedentary activity, affecting students’ health and leading to a low body mass and a high level of body fat. In relation to this finding, a distinction should be made between university esports players and professional esports players, as the latter have to train to perform better and, unlike university players, do not have to attend classes or sit for most of the day. Another aspect to highlight about BMI is the statistically significant difference (*p* = 0.04) between the players who participated in the study from Europe (24.70) and Latin America (25.52); this is in line with the statistics in population by region, which is evidenced by the results of the study by Walpole et al. [60], who reported that the percentage of the European adult population that is overweight is lower than that of Latin America (55.6% and 57.9%, respectively).

Another purpose of this study was to learn about the motivational orientations of esports players and to generate further theoretical knowledge, as recommended by Kim and Thomas [46], who highlighted that the motivation to compete can be beneficial in obtaining the identity of the esports player and help to maintain the professional career. Nevertheless, given the lack of literature on the theoretical model used in this study on motivational aspects in professional esports players [39], we proceeded to contrast the results obtained in this study with other research in different related areas.

In relation to the results obtained in the present research, professional esports players have an orientation to intrinsic and extrinsic motivation over amotivation, which is stronger in the group of players who self-reported low levels of physical activity; this could be explained by the fact that group dedicates more time to esports competition and training because they are professionals, helping to understand the results of Rudolf et al. [57], who gave significant relevance to the discovery of possible deficits in health-related physical training of esports players considering that they have similar requirements and challenges as traditional athletes [10]. This orientation is similar to the trend of other works that have investigated motivation in sport, considering and applying the same instrument [61,62,63]. On the other hand, and in relation to the region of origin of the study participants, no statistically significant differences were found between Latin America and Europe for the dimensions of intrinsic and extrinsic motivation. However, motivation was higher in Latin America (*p* = 0.02), which could be explained by the fact that in Europe, esports players have greater possibilities for professional development due to it being a more developed and stable industry in that area [5]; therefore, the esports environment in Latin America is still in an emerging stage, which has repercussions on all the actors involved in the industry.

Finally, Pearson’s correlation analysis reported a negative correlation between intrinsic motivation and METs (*r* = −0.158) and between extrinsic motivation and METs (*r* = −0.174). This could be explained by the results of the study by Lafre-nière et al. [64], who carried out a correlation between the frequency of playing and the motivation scale applied, with positive results in all of them. This shows that the higher the frequency of playing, and therefore the more time spent sitting, the higher the motivation of the player.

The discussion of this leads us to the conclusion that further improvement of PA measurement should be pursued in order to establish a consensus and thus make specific recommendations for professional and amateur esports players.

In relation to the limitations of the study, in most of the studies, physical activity time is recorded as a self-report, which is a subjective way of measurement; therefore, physical field tests are required to allow an objective measurement of PA levels in representative samples. In addition, people tend to significantly overestimate their activity levels [65].

On the other hand, esports developers should think about methodologies that stimulate the practice of PA as an enhancer and strengthener of healthy habits and performance improvements, as esports as an activity is not where moderate to vigorous PA is performed, but rather PA is undertaken in leisure time or during training periods.

## 5. Conclusions

Firstly, it is possible to conclude that the SMS is a reliable instrument to be applied to professional e-sports players. Secondly, from the results obtained in this study, we can conclude that the majority of professional e-sports players in the sample analysed had high and moderate levels of physical activity. In relation to their body composition, at least half of the subjects in the sample were of normal weight.

Finally, to the dimensions of motivation, high mean values were generally identified, with the exception of amotivation. In particular, statistically significant differences were found for the three groups of physical activity level, identifying higher levels of both intrinsic and extrinsic motivation for the group with low physical activity level. A regards the motivation dimension, this was low for the three groups, with no statistical differences.

## Figures and Tables

**Table 1:** Characteristics of the sample.

Variable	Range/Value	*n* (%)
Age	18–20	109 (41.9%)
	21–25	138 (53.1%)
	26–30	13 (5%)
Country	Spain	56 (21.5%)
	Germany	43 (16.5%)
	Sweden	44 (16.9%)
	Chile	23 (8.8%)
	Argentina	26 (10%)
	Brazil	42 (16.2%)
	Mexico	26 (10%)
Months as professional	<12 months	5 (1.9%)
	12–24 months	165 (63.5%)
	24–36 months	37 (14.2%)
	36–48 months	41 (15.8%)
	>48 months	12 (4.6%)
Esports	League of Legends	116 (44.6%)
	Counter Strake	86 (33.1%)
	Hearthstone	6 (2.3%)
	Call of Duty	18 (6.9%)
	FIFA	7 (2.7%)
	Clash Royale	22 (8.5%)
	Rocket League	5 (1.9%)
BMI	Underweight	1 (0.4%)
	Normal weight	128 (49.2%)
	Overweight	112 (43.1%)
	Obese	19 (7.3%)

**Table 2:** Levels of Physical Activity and Motivation.

Variable	Level	*n* (%)
Physical Activity	Low	19 (7.3%)
	Moderate	120 (44.2%)
	High	121 (48.5%)
	Type of Motivation	M/SD
Motivation	IM—to know	5.43 ± 0.70
	IM—accomplishment	5.30 ± 0.85
	IM—stimulation	5.64 ± 0.57
	Identified Regulation	5.53 ± 0.75
	Introjected Regulation	5.46 ± 0.73
	External Regulation	5.41 ± 0.64
	Amotivation	2.21 ± 0.48

**Table 3:** Dimensions of Motivation according to Physical Activity Levels.

Dimensions	Physical Activity Level Low	Physical Activity Level Moderate	Physical Activity Level Hight	*p*-Value
IM—to know	5.64 ± 0.709	5.47 ± 0.717	5.34 ± 0.679	0.147
IM—accomplishment	5.71 ± 0.71	5.29 ± 0.861	5.24 ± 0.850	0.087
IM—stimulation	5.90 ± 0.472	5.66 ± 0.579	5.56 ± 0.585	0.05 *
Total Intrinsic Motivation	5.75 ± 0.54	5.47 ± 0.60	5.38 ± 0.58	0.039 *
Identified Regulation	5.92 ± 0.736	5.56 ± 0.782	5.42 ± 0.690	0.024 *
Introjected Regulation	5.82 ± 0.731	5.50 ± 0.735	5.35 ± 0.714	0.024 *
External Regulation	5.84 ± 0.607	5.40 ± 0.665	5.33 ± 0.599	0.06 *
Total Extrinsic Motivation	5.86 ± 0.58	5.49 ± 0.62	5.37 ± 0.56	0.004 *
Amotivation	2.19 ± 0.586	2.21 ± 0.474	2.21 ± 0.481	0.978

Note: * *p* < 0.05.

**Table 4:** Motivation dimensions, BMI, METs, and sedentary time by region.

Dimensions	Europe	Latin American	*p*-Value
Intrinsic motivation	5.45 ± 0.59	5.45 ± 0.59	0.99
Extrinsic motivation	5.47 ± 0.60	5.47 ± 0.61	0.98
Amotivation	2.15 ± 0.47	2.29 ± 0.48	0.02 *
BMI Total	24.70 ± 3.05	25.52 ± 3.32	0.04 *
Total METs	2533.23 ± 2017.71	2428.55 ± 1437.69	0.63
Sedentary Behaviour	554.13 ± 116.78	583.08 ± 56.17	0.01 **

Note: * *p* < 0.05; ** *p* < 0.01.

**Table 5:** Correlation between dimensions of the SMS scale and socio-demographic variables.

	1	2	3	4	5	6	7	8	9
Intrinsic Motivation (1)	1	0.883 **	0.257 **	−0.047	−0.158 *	0.015	0.095	−0.101	−0.019
Extrinsic Motivation (2)		1	0.269 **	−0.041	−0.174 **	−0.017	0.078	−0.093	0.022
Amotivation (3)			1	0.009	−0.014	−0.014	−0.026	−0.029	−0.022
Age (4)				1	0.043	0.685 **	0.052	−0.018	−0.031
METs (5)					1	0.006	−0.147 *	0.060	0.081
Professional Months (6)						1	0.096	0.035	0.014
Sedentary Behaviour (7)							1	0.016	0.004
Weight (8)								1	0.383 **
Size (9)									1

Note: * Correlation is significant at the 0.05 level; ** correlation is significant at the 0.01 level.

## Data Availability

Not applicable.
